# Deep learning-assisted PET imaging achieves fast scan/low-dose examination

**DOI:** 10.1186/s40658-022-00431-9

**Published:** 2022-02-04

**Authors:** Yan Xing, Wenli Qiao, Taisong Wang, Ying Wang, Chenwei Li, Yang Lv, Chen Xi, Shu Liao, Zheng Qian, Jinhua Zhao

**Affiliations:** 1 Department of Nuclear Medicine, Shanghai General Hospital, Shanghai Jiaotong University, No. 100 Haining Road, Shanghai, 200080 People’s Republic of China; 2 United Imaging Healthcare, Shanghai, People’s Republic of China; 3 Shanghai United Imaging Intelligence Co. Ltd, Shanghai, People’s Republic of China

**Keywords:** Positron emission tomography and computed tomography (PET/CT), Deep learning, Denoising technique, Image quality

## Abstract

**Purpose:**

This study aimed to investigate the impact of a deep learning (DL)-based denoising method on the image quality and lesion detectability of ^18^F-FDG positron emission tomography (PET) images.

**Methods:**

Fifty-two oncological patients undergoing an ^18^F-FDG PET/CT imaging with an acquisition of 180 s per bed position were retrospectively included. The list-mode data were rebinned into four datasets: 100% (reference), 75%, 50%, and 33.3% of the total counts, and then reconstructed by OSEM algorithm and post-processed with the DL and Gaussian filter (GS). The image quality was assessed using a 5-point Likert scale, and FDG-avid lesions were counted to measure lesion detectability. Standardized uptake values (SUVs) in livers and lesions, liver signal-to-noise ratio (SNR) and target-to-background ratio (TBR) values were compared between the methods. Subgroup analyses compared TBRs after categorizing lesions based on parameters like lesion diameter, uptake or patient habitus.

**Results:**

The DL method showed superior performance regarding image noise and inferior performance regarding lesion contrast in the qualitative assessment. More than 96.8% of the lesions were successfully identified in DL images. Excellent agreements on SUV in livers and lesions were found. The DL method significantly improved the liver SNR for count reduction down to 33.3% (*p* < 0.001). Lesion TBR was not significantly different between DL and reference images of the 75% dataset; furthermore, there was no significant difference either for lesions of > 10 mm or lesions in BMIs of > 25. For the 50% dataset, there was no significant difference between DL and reference images for TBR of lesion with > 15 mm or higher uptake than liver.

**Conclusions:**

The developed DL method improved both liver SNR and lesion TBR indicating better image quality and lesion conspicuousness compared to GS method. Compared with the reference, it showed non-inferior image quality with reduced counts by 25–50% under various conditions.

**Supplementary Information:**

The online version contains supplementary material available at 10.1186/s40658-022-00431-9.

## Introduction

Positron emission tomography and computed tomography (PET/CT) is a non-invasive imaging modality widely used in oncology, providing both anatomical and functional information. In oncology, PET/CT is a powerful tool for diagnosis, cancer staging and re-staging, radiation therapy planning, prognosis, and treatment‐response monitoring [1–5].

To provide diagnostic PET images with sufficient image quality, a certain activity administered to the patient combined with an adequate acquisition time is recommended in the guideline for oncological ^18^F-FDG PET imaging [1]. As radiation exposure is always a concern for both patients and operators, especially in radiation-sensitive populations [6]. In addition, the reduction on the administered activity is expected in the clinical management on the patients including those who need multiple PET examinations to monitor the therapy response [7–9]. Over the years, with the advent of advanced PET/CT scanners and image reconstruction algorithms such as time of flight (TOF), activity reduction is possible in pre-clinical studies and clinical practice [10–12]. However, the reduced injected activity/acquisition time always causes increased noise, lower signal-to-noise ratio (SNR), and potentially unnecessary artefacts in PET images.

Many post-processing techniques have been employed to reduce PET image noise, such as Gaussian filtering, Metz filtering [13], wavelet transform [14, 15], non-local mean [16, 17] and BM3D [18]. In general, the noise distribution is modelled in either the transform domain or the spatial domain, and a dedicated filter or a chain of well-designed filters are used to remove the noise. The mathematics behind these conventional methods are explicit, and only a few parameters are involved in the whole process. Thus, these methods are of limited performance in compromising the image noise and lesion detectability. Recently, deep learning methods have achieved great successes in image recognition, segmentation, registration, super-resolution, and image de-noising [19–22]. So far, three commercialized deep learning-based algorithms, designed to reduce the noise in PET images, have been approved by the U.S. Food and Drug Administration (FDA). They are SubtlePET (Subtle Medical), AiCE DLR (Canon Medical Systems Corporation), and HYPER DLR (United Imaging Healthcare).

HYPER DLR has been implemented in more than 10 sites worldwide where various clinical demands emerged according to their specific situations. Undoubtedly, a reduced injected activity is of great importance to reduce the radiation exposure to improve the patient care. On the other hand, due to the limited number of PET/CT scanners per unit population in China and other developing countries, the patient’s throughput of the department was large. Thus, a reduced PET acquisition time is expected to reduce the work burden while maintaining an acceptable image quality. In addition, the algorithm has also been used on the mobile PET/CT scanners whose working time for a certain place was critical. Previous studies have been conducted to evaluate the image quality and lesion detectability of commercialized deep learning algorithms with clinical cases [23, 24]. In this study, the nuclear medicine physicians identified all the suspected lesions as in the clinical practice Furthermore, a detailed analysis on the lesion and patient characteristics, including the patient body mass index (BMI), lesion size and uptake, was performed to comprehensively investigate the performance of the proposed deep learning method. This study aimed to investigate both the image quality and lesion detectability of HYPER DLR with standard and reduced administered activity in comparison to the Gaussian filter in ordered subset expectation maximization (OSEM) approach. Moreover, several subgroup analyses based on different categories were performed to investigate the performance and limitation of its application in the oncological studies.

## Methods

### Network structure

**Fig. 1:**
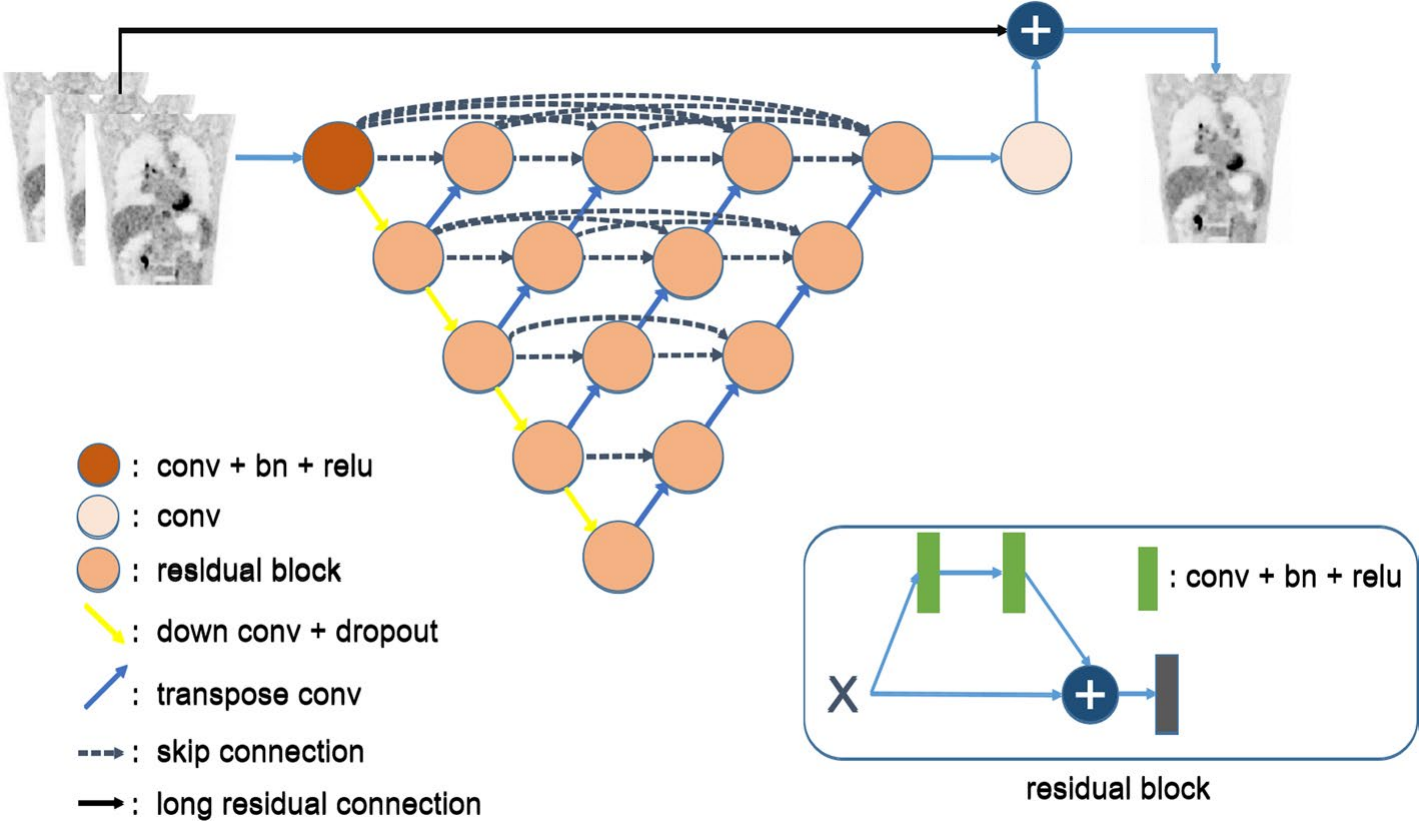
Network architecture of HYPER DLR. relu: An activation function defined as the positive part of its argument: *f*(*x*) = max(0,*x*), where *x* is the input to a neuron. conv: Convolution is the most basic operation in convolutional neural networks. The input image is convolved with the network to extract features in the image. bn: A technique for improving speed, performance, and stability of the artificial neural network. It is used to normalize the input layer by adjusting and scaling the activations. dropout: A regularization technique for reducing overfitting in neural networks by preventing complex co-adaptations on training data. skip connection: A technique to help solve the problem of vanishing gradients, allowing faster training. It builds shortcuts to jump over some layers

The network used in HYPER DLR is based on the UNet architecture, and adopts the techniques of residual connection and dense connection used in the ResNet [25] and the DenseNet [26]. The structure of the proposed network is shown in Fig. 1. The top layer of the network is a long residual connection from the input to the output, which allows the network to learn the residual, i.e. the noise component, between the target image and the input image and accelerates the convergence of the deep network. From top to bottom, the upper layer has a larger dimension and higher resolution, and a dense connection is used to reduce the loss of the information. By contrast, a sparse connection is adopted in the lower layer. The network uses a large number of residual blocks which can solve the problem of gradient disappearance while improving the performance and ensuring the convergence of the deep network.

### Network training and validation

In this network, a total number of 313 studies were used for training and another 80 studies were used for validation. The data were obtained from 4 sites where the PET/CT scanners manufactured by UIH have been installed. The patient age ranged from 18 to 90 years, with a median of 55 years. The injected dose was 3.1–4.3 MBq/kg, while the acquisition time was 90–180 s/bed for the body part. The retrospective PET reconstruction with 50% acquisition time was used as a training input, i.e. high-noise PET images. The retrospective reconstruction with full acquisition time as the training target, i.e. low-noise PET image. Longer acquisition time results in better training target images, and may further improve the performance of the network. All images were reconstructed with OP-OSEM algorithm incorporating TOFand point spread function (PSF). The iteration number and the subsets were fixed at 2 and 20, respectively. The voxel size of the training image was 2.34 × 2.34 × 2.68 mm. In order to further improve the robustness of the network and reduce the effect of overfitting, data augmentation techniques such as horizontal and vertical flips were also used. All training images were resampled such that the images had the same size and resolution. The image intensity was normalized within the range [0, 1]. A 2.5D processing was done for the normalized PET images. That is, five slices of the input images correspond to one slice of the target image. More specifically, the network uses the image patches with a dimension of 64 × 64 × 5 as the training input and the image patches with a dimension of 64 × 64 × 1 as the training target. During the training process, L1 Loss function was adopted. The model was trained with the Adaptive Moment Estimation (ADAM) optimizer. The initial learning rate was set to 10^–4^ and then halved after 20 epochs. The batch size used to train the network was set to 32, and the total number of epochs to train the network was set to 200. Our network was implemented in the PyTorch framework and Python 3.7. The testing was performed on a computer with one Quadra P5000 GPU. The CUDA library was 8.0 and cuDNN version was 7.0.5.

### Patients

The study included 52 consecutive patients (female/male: 19/33, age: 24–87 years) with known or suspected malignancies who were referred to the Shanghai General Hospital from 3 to 19 November 2020 for clinical ^18^F-FDG PET/CT examinations. Their demographic and clinical data are listed in Table 1. All patients had fasted for at least 6 h prior to the PET/CT scans and a blood glucose level of ≤ 10 mmol/mL was confirmed by finger-prick sampling. A weight-based ^18^F-FDG dose (259 ± 48 MBq, range: 188–407 MBq) was administered as an intravenous bolus to the patient. Patients were instructed to stay in a warm environment and drink 0.5–1.0 L water during the uptake. The uptake time was 66 ± 12 min (range: 45–97 min). This retrospective study was approved by the Institutional Review Board of Shanghai General Hospital, and the need for written informed consent was waived.

**Table 1:** Patient demographic characteristics

Parameter	Value
Age (years)	62.7 ± 14.0 [24, 87]^a^
Weight (kg)	65.6 ± 11.8 [46, 110]^a^
Height (cm)	165.00 ± 8.79 [150, 184]^a^
BMI	23.9 ± 3.3 [16.3, 32.5]^a^
Injected activity (MBq)	259 ± 48 [188, 407]^a^
Uptake time (min)	66 ± 12 [45, 97]^a^
*Primary cancer type*	
Leukaemia	1^b^
Nasopharyngeal cancer	2^b^
Gallbladder cancer	2^b^
Multiple myeloma	1^b^
Lung cancer	8^b^
Liver cancer	1^b^
Cervical cancer	3^b^
Laryngeal carcinoma	2^b^
Colon cancer	4^b^
Colorectal cancer	1^b^
Lymphoma	7^b^
Ovarian cancer	2^b^
Bladder cancer	2^b^
Renal cancer	2^b^
Adrenocortical carcinoma	1^b^
Oesophageal cancer	3^b^
Gastric cancer	1^b^
Pancreatic cancer	4^b^
Rectal cancer	2^b^
Endometrial cancer	3^b^

BMI, body mass index

^a^Data are presented as the mean ± standard deviation [minimum, maximum]

^b^Number of patients

### PET/CT imaging

Images were acquired on a digital PET/CT scanner (uMI 780, United Imaging Healthcare, China), configured with a 30-cm axial field of view. A CT scan was performed prior to PET imaging for attenuation correction and anatomical localization with a fixed tube voltage of 120 kV and an auto-mAs technique for dose modulation. Subsequently, a whole-body PET scan was performed from the skull to the mid-thigh. Data were acquired in a 3D list-mode with 180-s acquisition for each bed position and auto-adjusted overlap (25–50%).

PET raw data were reconstructed with four different acquisition durations (180, 135, 90, and 60 s) by rebinning the list-mode data to simulate reduced injected activity. Two post-processing methods, Gaussian filter with full width at half maximum of 3 mm and HYPER DLR, were applied to each dataset. For each patient, eight series of images were generated, referred to as GS_180, GS_135, GS_90, GS_60, DLR_180, DLR_135, DLR_90, and DLR_60 groups. For the OSEM algorithm, reconstruction was performed with two iterations, 20 subsets, 192 × 192 matrix, 600 field of view, as well as TOF and PSF. For HYPER DLR, a body model was selected for the whole-body reconstruction instead of the Gaussian filter, while all other reconstruction parameters in the OSEM algorithm remained the same as those in the above-mentioned GS method. All PET reconstructions included standard corrections like decay, scatter, random, dead time, attenuation, and normalization.

### Image analysis

#### Qualitative analysis

**Fig. 2:**
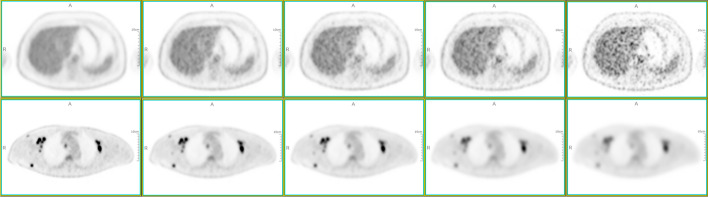
Illustration of the scores from 5 to 1 (from left to right) in two perspectives. Overall image noise (upper row). Lesion contrast (bottom row)

The PET image quality was assessed by two nuclear medicine physicians each with more than ten years of experience in interpreting PET/CT images. For each patient, the reading order of the 8 series of PET images was randomized by an independent operator. In addition, the patient’s history, as well as the acquisition duration and reconstruction algorithm, were blinded to the reader. A 5-point Likert scale was used for the independent assessment of the image quality in two perspectives: lesion contrast and overall image noise. A score of 1 rated images with excessive noise and unfavourable lesion contrast. Images with sub-optimal noise and lesions with blurring leading to impaired diagnostic confidence were rated with a score of 2. A score of 3 was given if image noise and lesion contrast were substantially equivalent to those in routine oncological ^18^F-FDG PET/CT images. A score of 4 indicated preferred image noise and good lesion contrast with diagnostically irrelevant blurring, whereas images with optimal noise and clear lesion contrast were rated with a score of 5. Figure 2 shows reference images with different Likert scores. The image quality scores independently assessed by the two raters were documented for the inter-reader agreement test.

In addition, the number of ^18^F-FDG-avid lesions in each PET series was determined to assess the lesion detectability. The GS_180 group was used as a reference. If the result of the two physicians was not consistent, the result was determined in a consensus meeting held by a third physician experienced in nuclear medicine.

#### Semiquantitative analysis

Semiquantitative analyses were performed on a dedicated workstation (uWI, United Imaging Healthcare, China). A region of interest (ROI) with a diameter of 30 ± 3 mm was manually drawn at the same position and slice on a homogeneous area of the right liver lobe. The SUVmean, SUVmax, and standard deviation (SD) values of the liver for each series were recorded. The liver SNR, as a measure of image quality, was obtained by dividing the SUVmean by its SD. For identified FDG-avid lesions, SUVmax was recorded by drawing a ROI on the transverse slice with the maximum diameter. Furthermore, the target-to-background ratio (TBR) for each identified lesion, calculated by dividing the lesion SUVmax by the liver SUVmax, was used as a measure of image contrast. The diameters of the lesions were measured on the CT images for further investigation. For any missed lesion that cannot be identified in the image interpretation, a small ROI with a diameter of around 10 mm was carefully drawn adjacent to the lesion to measure the background uptake. The tumour-to-surrounding-background ratio (TsBR) was additionally obtained by dividing the lesion SUVmax by the SUVmax of the surrounding background.

### Statistical analysis

Continuous parameters are presented as the mean ± SD and range. Inter-reader agreement was evaluated using Cohen’s kappa test. The subjective scores of the image quality were subsequently compared using the Wilcoxon signed-rank test. All parameters in the semiquantitative analysis were tested for normality using the Kolmogorov–Smirnov test. Bland–Altman plot analyses were performed to assess the agreement of the SUVs between the GS and DLR groups. Concordance correlation coefficient (CCC) and linear regression were used to quantify the agreement on SUVs between groups. Subsequently, the two-tailed paired samples *t*-test was performed to investigate the differences in liver SNR, SD, and lesion TBR between groups. Furthermore, subgroup analyses were performed using the paired samples *t*-test to compare lesion TBRs between groups. Statistical significance was considered for a p-value less than 0.05, and all statistical tests were performed using SPSS Statistics, version 25 (IBM, Armonk, NY, USA) and Microsoft Excel.

## Results

### Qualitative image quality

**Fig. 3:**
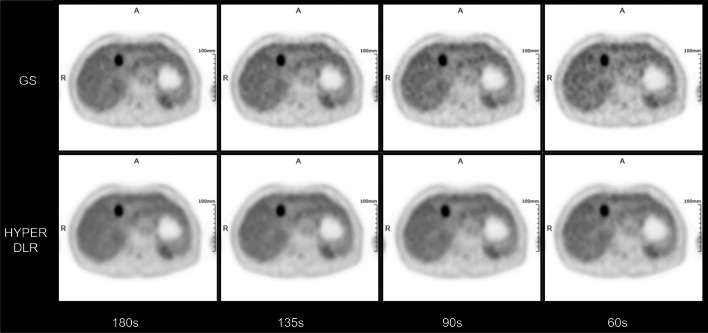
Transverse images of the liver metastasis of an 80-year-old male patient with renal cancer. The upper and bottom row were reconstructed with GS and HYPER DLR approach, respectively. The acquisition time is 180, 135, 90, and 60 s (left to right). The bottom row shows the superior performance in noise reduction

**Fig. 4:**
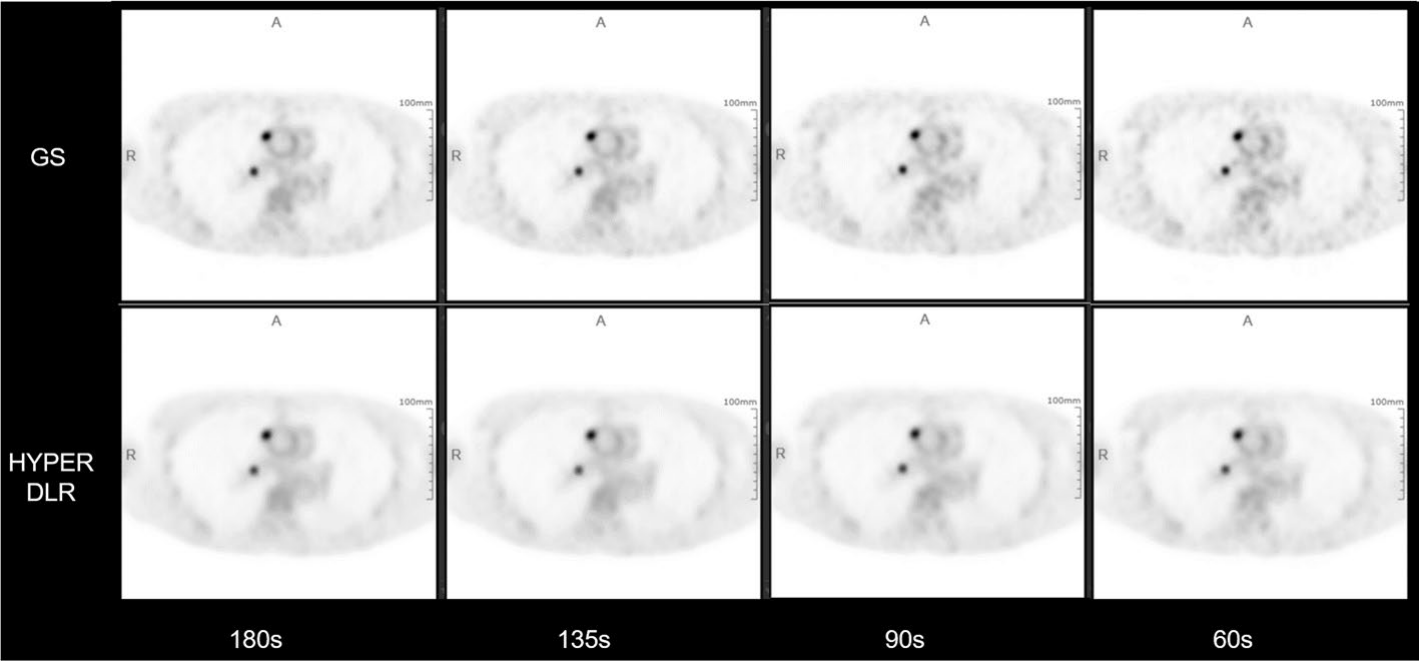
Transverse images showing lymph node metastasis of a 61-year-old female patient with colorectal cancer. The upper and bottom row were reconstructed with GS and HYPER DLR algorithm, respectively. The acquisition time is 180, 135, 90, and 60 s (left to right). The bottom row indicates a performance equivalent to the one in the upper row regarding lesion detection with decreased image noise in the surrounding tissue

The overall inter-reader agreement regarding lesion contrast and image noise showed kappa values of 0.705 and 0.913, indicating excellent agreement between the readers. The average scores of the two readers for each GS and DLR group are listed in Table 2. Images in all DLR groups showed significantly suppressed image contrast and reduced image noise compared to the reference GS_180 group (all *p* < 0.001). When comparing GS and DLR images with the same acquisition time, DLR method yielded more smoothed images with less noise (*p* < 0.05, Figs. 3 and 4). Moreover, all images of the DLR_180 group were rated with a score of 5 by both physicians, demonstrating the superior performance of HYPER DLR in noise reduction.

**Table 2:** Qualitative image quality scores

	Contrast	Noise
GS_180	4.62 ± 0.53	3.81 ± 0.44
GS_135	4.64 ± 0.52	3.70 ± 0.50
GS_90	4.65 ± 0.52	3.28 ± 0.49
GS_60	4.38 ± 0.83	2.54 ± 0.54
DLR_180	4.28 ± 0.65	5.00 ± 0.00
DLR_135	4.26 ± 0.70	4.97 ± 0.17
DLR_90	4.13 ± 0.66	4.77 ± 0.45
DLR_60	4.00 ± 0.19	4.13 ± 0.41

**Fig. 5:**
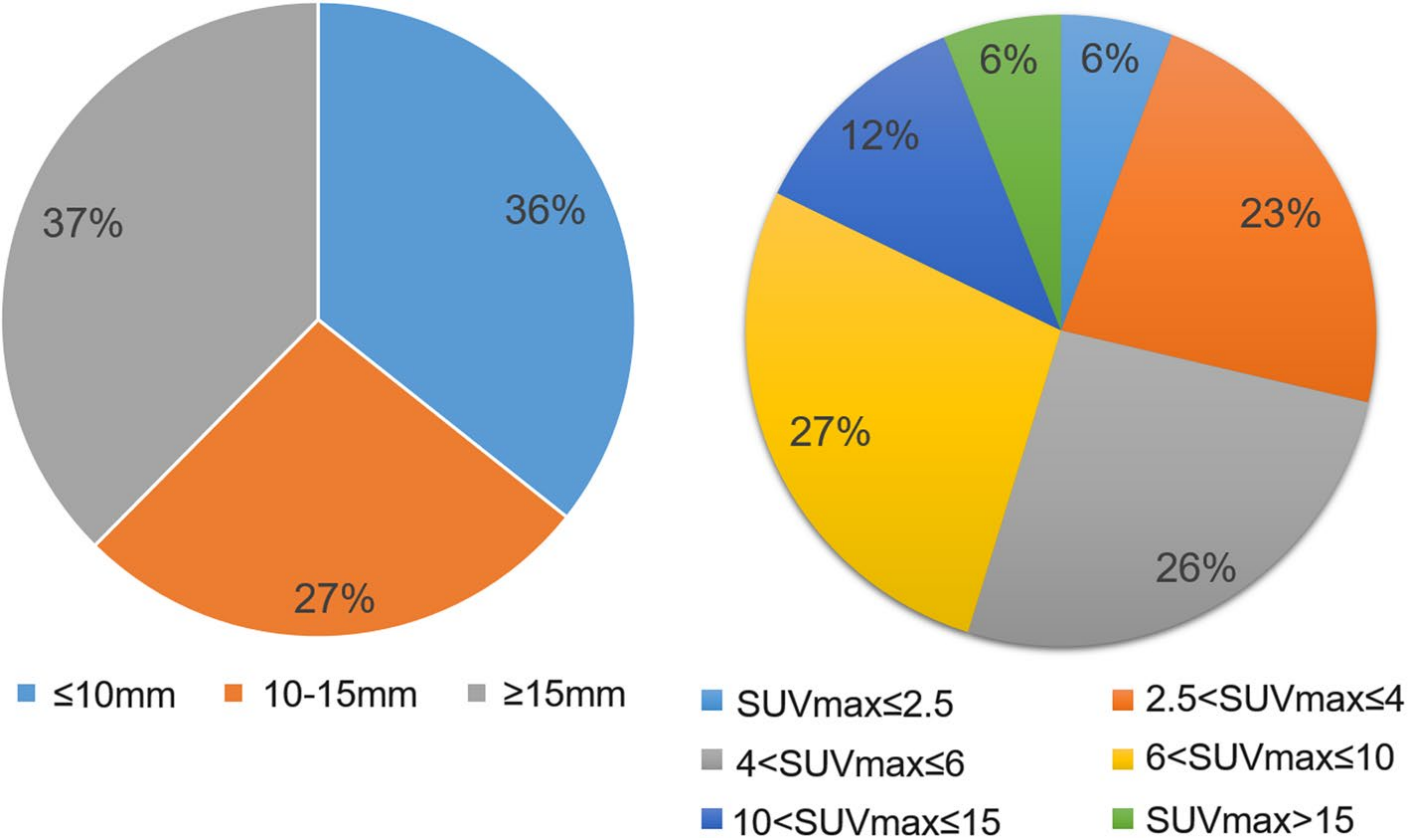
Characteristics of the identified lesions (*n* = 314). Left: Diameters of the lesions measured in computed tomography images. Right: SUVmax of the lesions measured in the reference GS_180 PET images. SUV, standardized uptake value

A total number of 314 FDG-avid lesions were identified in the GS_180 group and used as a reference (Fig. 5). For the DLR groups, more than 96.8% of the lesions were successfully identified. The DLR_180 and DLR_135 groups had the best performances where only two ^18^F-FDG-avid lesions were missed. Both lesions were overlooked due to an uptake similar to that of the surrounding tissue, with a low TsBR value ranging from 1.09 to 1.18. In the DLR_90 group, additional two lesions were not detected by the readers. The DLR_60 group showed the worst performance among the 8 groups regarding lesion detection with 10 missed lesions. All missed lesions had a diameter of less than 10 mm. However, the missed lesions had no impact on the cancer staging. In subsequent analyses at the location of the missed lesions, we found that all lesions were metastases, including metastatic lymph nodes and bone and liver metastases. Moreover, all missed lesions had uptake values similar to those of the surrounding tissues. The signals of these metastases were also suppressed although the image noise of the surrounding tissue was reduced.

### Semiquantitative image quality

**Fig. 6:**
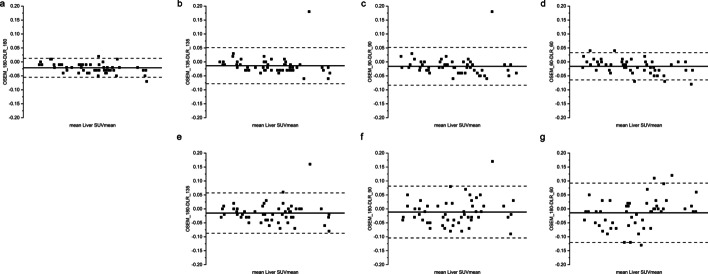
Bland–Altman plots of liver SUVmean between GS and DLR groups. Subfigures **a-d **demonstrate the agreement of liver SUVmean between GS and DLR images with the same acquisition durations. Subfigures **a**, **e**, **f** and **g** demonstrate the agreement of liver SUVmean between DLR groups and the reference GS_180 group. The above plots show minimal differences in liver SUV between groups. SUV, standardized uptake value

**Fig. 7:**
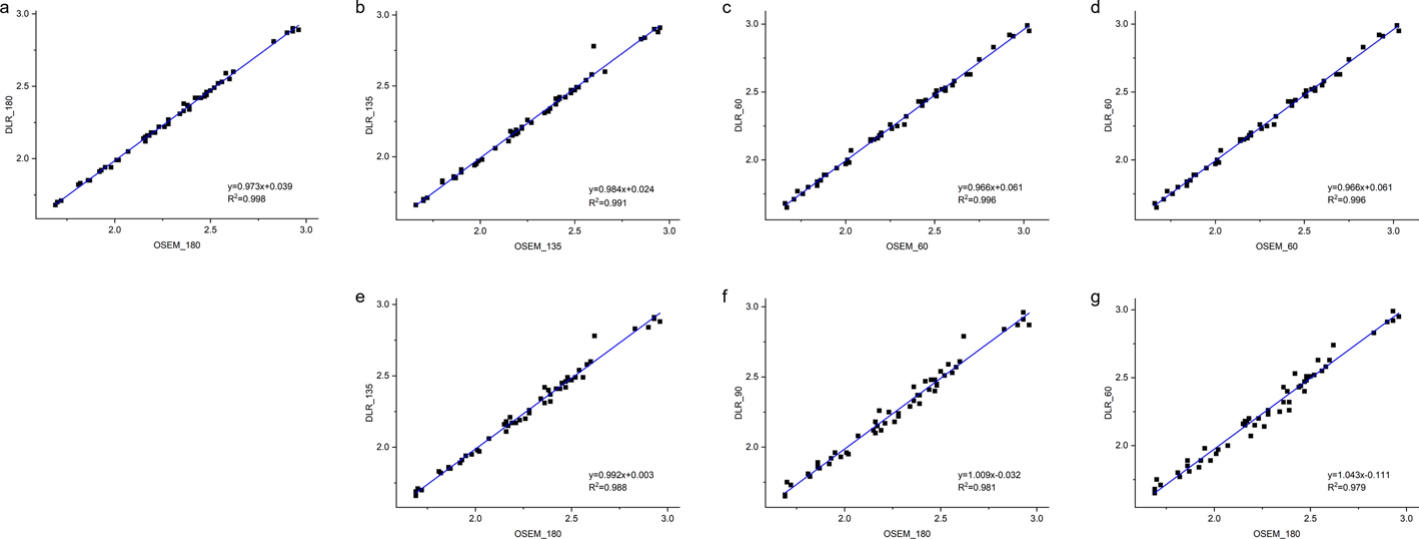
Linear regression of liver SUVmax between GS and DLR groups. Subfigures **a-d** show the results between GS and DLR images with the same acquisition time. Subfigures **a**, **e**, **f** and **g** indicate a strong relationship between DLR groups and the reference GS_180 group. The above results demonstrate the good agreement of SUV between the groups. SUV, standardized uptake value

**Fig. 8:**
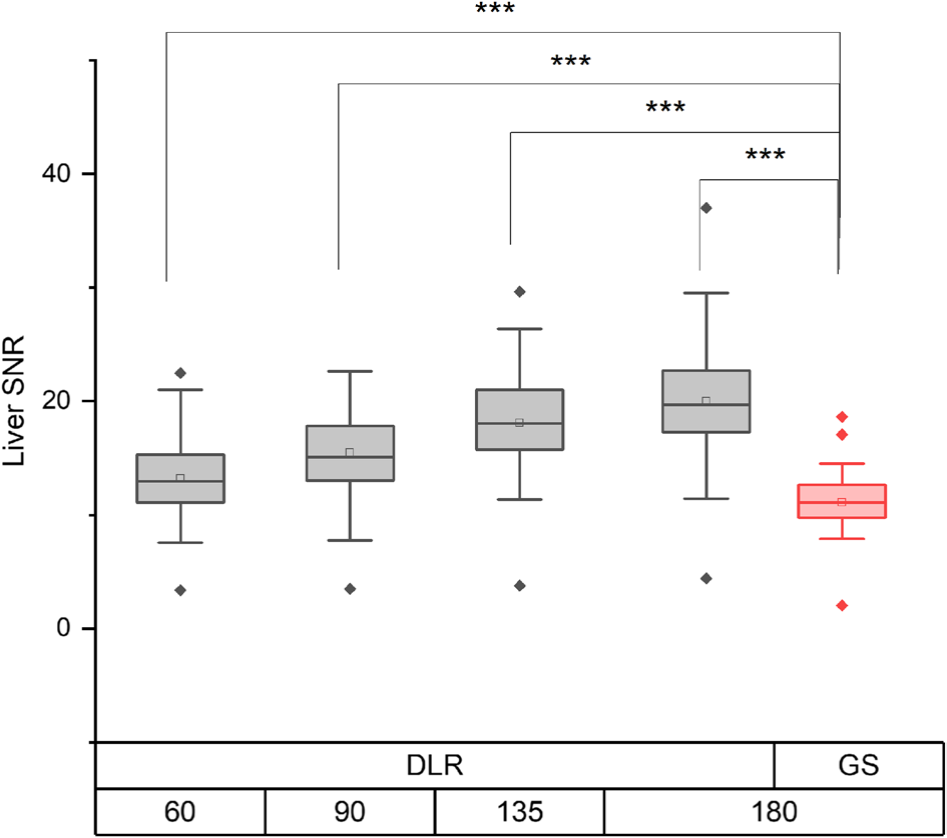
Comparison of the liver SNR between DLR groups and the referenceGS_180 group. All DLR groups show a significantly improved liver SNR compared to the reference group, indicating reduced image noise. ***p < 0.001. SNR, signal-to-noise ratio

The liver SUVmean between groups agreed well as shown in Bland–Altman plots (Fig. 6). The CCCs of the liver SUVmean were all larger than 98.7% for the DLR groups compared to the GS groups, as shown in Table 3, demonstrating very strong agreement between the DLR and GS groups (Fig. 7). Because the HYPER DLR algorithm was able to suppress image noise, the liver SNR in the DLR groups was significantly higher than that in the reference GS_180 group. In this study, even the DLR_60 group showed a significantly higher SNR value than the reference GS_180 group (Fig. 8).

**Table 3:** Differences in SUV of liver and lesion tissues

Parameter	Comparison group A–B	Pearson coefficient *R*^2^	CCC
Liver SUVmean	GS_180-DLR_180	0.998	0.997
	GS_180-DLR_135	0.991	0.993
	GS_180-DLR_90	0.990	0.990
	GS_180-DLR_60	0.996	0.987
	GS_135-DLR_135	0.988	0.995
	GS_90-DLR_90	0.981	0.994
	GS_60-DLR_60	0.979	0.997
Lesion SUVmax	GS_180-DLR_180	0.994	0.991
	GS_180-DLR_135	0.991	0.989
	GS_180-DLR_90	0.984	0.985
	GS_180-DLR_60	0.975	0.977
	GS_135-DLR_135	0.993	0.989
	GS_90-DLR_90	0.990	0.985
	GS_60-DLR_60	0.986	0.978

CCC, concordance correlation coefficient; SUV, standardized uptake value

**Fig. 9:**
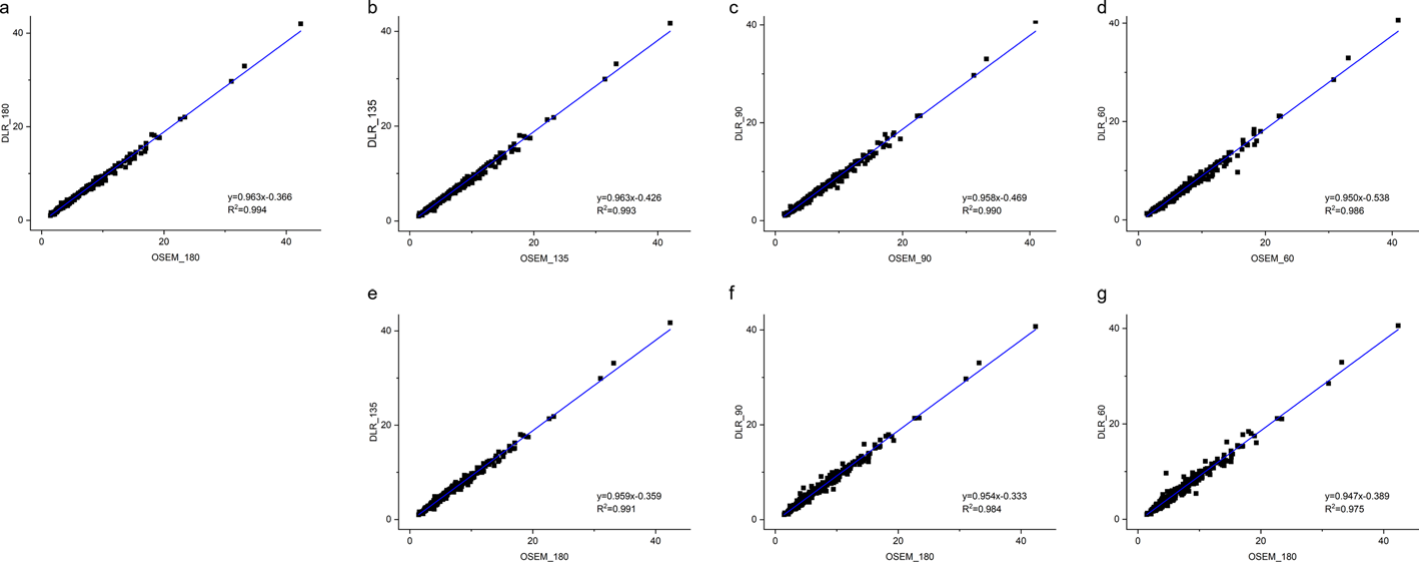
Linear regression of lesion SUVmax between GS and DLR groups. Subfigures **a**–**d** show the results between GS and DLR images with the same acquisition time. Subfigures **a**, **e**, **f** and **g** indicate a strong relationship between DLR groups and the reference GS_180 group. The above results demonstrate the good agreement of SUV between the groups. SUV, standardized uptake value

**Fig. 10:**
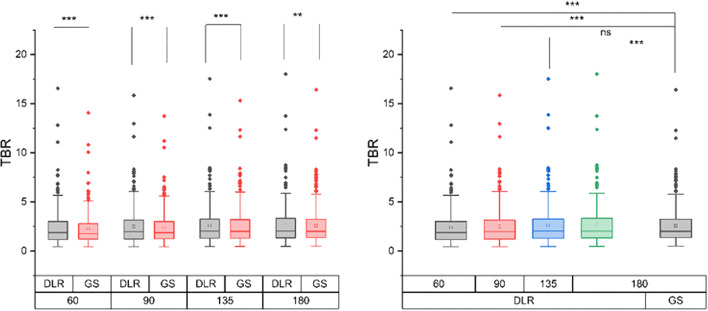
Comparison of the lesion TBR between GS and DLR groups. Left: The HYPER DLR algorithm can improve the lesion contrast significantly compared to that achieved by GS approach with the same acquisition duration. Right: Compared with the reference GS_180 group, the DLR_135 group shows no significant difference in TBR, indicating a similar lesion contrast. **p < 0.01; ***p < 0.001; ns, no significant difference. TBR, target-to-background ratio

The equivalent tests comparing lesion SUVmax between GS and DLR groups are presented in Table 3. The CCCs of the lesion SUVmax demonstrated a very strong agreement between the GS and DLR groups with all values higher than 97.7% (Table 3 and Fig. 9). The DLR groups also showed a better performance in the TBR compared to the corresponding GS group with the same acquisition time (Fig. 10). There was no significant difference between the TBR values of the DLR_135 group and the reference GS_180 group (*p* = 0.713), demonstrating its improvement on lesion contrast with lower counts. Both DLR_90 and DLR_60 groups showed a significantly lower TBR value than the reference (Fig. 10).

### Subgroup analyses

Subsequently, a subgroup analysis was performed with different parameters. To investigate the performance of HYPER DLR regarding image contrast, a subgroup analysis was performed by dividing the lesions into two groups based on the lesion diameter measured on diagnostic CT images: a small lesion group with diameters of less than 15 mm and a large lesion group with diameters of at least 15 mm [27]. In the small lesion group (*n* = 196), the TBR was not significantly different between the DLR_180 group and the reference GS_180 group (*p* = 0.051) whereas the TBR values in other DLR groups were significantly lower than that in the reference GS_180 group (all *p* < 0.001). In the large lesion group (*n* = 118), the TBR values in the DLR_180 and DLR_135 groups were significantly improved compared to that in the reference GS_180 group (*p* < 0.001 and *p* = 0.028, respectively), and there was no significant difference between the DLR_90 group and the reference group (*p* = 0.19). This indicates that for these relatively larger lesions, the HYPER DLR algorithm has a better contrast enhancement.

In addition, we further divided the lesions using another threshold since all missed lesions had a diameter of less than 10 mm: a small lesion group with a diameter below 10 mm and a large lesion group with a diameter of 10 mm or above. In the small lesion group (*n* = 112), the TBR values of all DLR groups were significantly lower than that of the reference GS_180 group (all *p* < 0.001). However, in the large lesion group (*n* = 202), there was no significant difference in TBR between the DLR_135 group and the reference GS_180 group.

In a subsequent subgroup analysis, the lesions were divided based on whether their mean uptake value was lower than the liver uptake in the reference group. Group G1 comprised lesions whose uptake values were lower than the liver uptake values whereas all other lesions were classified as Group G2. Group G1 (*n* = 37) revealed a limitation of the HYPER DLR algorithm for lesions with lower uptake because the TBR values in all DLR groups were significantly lower than that in the reference GS_180 group (all *p* < 0.001). In Group G2 (*n* = 277), there was no significant difference between the DLR_135 group and the reference GS_180 group (*p* = 0.208).

Finally, a subgroup analysis was performed based on patient habitus. The image quality of overweight or obese patients is decreased according to a previous study [28]. In our subgroup analysis, the enrolled patients were according to WHO criteria divided into a Group B1 with a body mass index (BMI) of less than 25 kg/m^2^ and a Group B2 with a BMI of at least 25 kg/m^2^. In Group B1 with a total number of 220 lesions, the DLR groups showed a significant improvement in TBR compared to the corresponding GS groups with the same acquisition time. However, there was no significant difference between the DLR_135 group and the reference GS_180 group (*p* = 0.501). In Group B2 with a total number of 94 lesions, there was no significant difference between the reference GS_180 group and the DLR_180, DLR_135, and DLR_90 groups (*p* = 0.094, 0.685, and 0.098, respectively). These findings suggest that the HYPER DLR algorithm has the capability of improving the lesion contrast in images of inferior quality.

## Discussion

Deep learning-based denoising algorithms provide an effective solution for maintaining image quality with shortened acquisition time or reduced injected activity. Studies have been performed to evaluate the performance of deep learning-based methods either approved by authority organization that can be implemented in clinical use [23, 24] or still within the research field at different noise levels [29–34]. The performance of the algorithms depends on the network structure, the training strategy and the training dataset. In a recent real-world low-dose PET study on a deep learning-based denoising technique, the patients were injected with a reduction of 18F-FDG by 33%, and it demonstrated the non-inferiority of AI processed image quality and lesion detectability compared with the standard method [23]. Moreover, this study included an assessment of a business case. In another study with a deep learning enhancement (DLE) model, images reconstructed with block sequential regularised expectation maximisation (BSREM) was used as the target images during the training. This study compared the results of DLE methods for different acquisition time with the full-duration BSREM method and found the models may allow a reduction in acquisition time/injected activity by 50% [34]. Our study lacked such a business case assessment, and used the standard OSEM images during the network training. However, subgroup analyses on the lesion detectability and the characteristics of the enrolled patients, lesions (size and uptake) were performed to comprehensively evaluate the performance and limitation of the HYPER DLR method. In our study, the enrolled patients were from an independent site and presented various conditions regarding age, weight, height, and cancer type to provide a heterogeneous sample reflecting common clinical practice. Short-duration datasets were reconstructed with the OSEM and the proposed HYPER DLR method, of which the image quality and lesion detectability were compared. In our clinical evaluation, the HYPER DLR method had a superior capability for noise reduction compared to the GS method from both subjective and semiquantitative perspectives. Moreover, it can improve the image quality with a shorter acquisition time, indicating its potential in studies with lower administered activity. According to our study results, the administered activity can be reduced by 25% without compromising quantitative SUV and TBR values compared to the standard dose.

In addition, the HYPER DLR algorithm can provide better performance in patients with higher BMI. It is well-known that the PET image quality deteriorates with increasing patient weight for linear weight-based ^18^F-FDG dose regimens [26]. However, our study demonstrates that the administered activity in overweight and obese patients can even be reduced by 50% without compromising image quality as compared to the standard FDG administration. This will be helpful in reducing the total activity administered to overweight and obese patients and will reduce radiation exposure-induced risks for patients and operators [35, 36].

In oncology, lesion detectability is important in the early diagnosis and staging of patients. Early detection of lesions can lead to a positive patient prognosis, so the ability to detect small, low-intensity/uptake lesions is of high importance and was evaluated in the study. HYPER DLR was able to maintain a lesion detectability over 98.6% even if the acquisition time was reduced by 66.7% of that during standard acquisition. If the acquisition time was only reduced by 25%, the lesion detectability can be up to 99.4%, with only two lesions missed in our study. These two lesions were adjacent to the aorta and the scalp, respectively, where the physiological uptake of the surrounding tissues was relatively high. After checking the uptake of the missed lesions, the maximum SUVmax was up to 3.64, measured in a liver metastasis of a 74-year-old female with gallbladder carcinoma (Additional file 1: Fig. S1). In both DLR_90 and DLR_60 images, this metastasis was overlooked due to high image noise and respiratory motion artefacts. Moreover, the liver was found to have a relatively high uptake which may also have impacted the identification of this metastasis. Similarly, all missed lesions had uptake values approximately comparable with those of the surrounding tissues, with TsBR values from 1.004 to 1.595. We further investigated the lesion with the maximum TsBR value and found it to be a pelvic metastatic lymph node adjacent to the intestinal tract (Additional file 2: Fig. S2). It was challenging to distinguish this small lesion with a diameter of 6.1 mm from the physiological uptake observed in the adjacent intestinal tract. The missed lesions had an average diameter of 8.6 ± 1.3 mm (range: 6.1–10.0 mm). According to our subgroup analysis, TBR values, as a measure of contrast, of small lesions with a diameter of less than 10 mm were suboptimal compared to those in GS groups. This may impede the lesion identification in HYPER DLR PET images.

The subjective assessment of the image quality performed by nuclear medicine physicians demonstrated the superior noise reduction performance of the HYPER DLR algorithm. Even if the acquisition time was reduced by 66.7% in the HYPER DLR group, the performance of this group regarding image noise was superior to that of the reference GS_180 group. The noise reduction in PET images can help physicians better interpret PET/CT images where false-positive uptakes were reduced. However, regarding lesion contrast, the subjective assessment showed a better performance of the GS method compared to the HYPER DLR algorithm which was not consistent with the later semiquantitative analysis in this study. This bias may mainly be due to the long-term experience in using the GS method. The subjective assessment might improve when physicians get used to the assessment of HYPER DLR images.

Deep learning methodologies, although achieved superior performance against conventional methods, still have room to improve. Firstly, the network architecture should be re-designed. For example, using a fully 3D architecture instead of 2.5D or incorporating the attention mechanism into the network. Secondly, a large and high quality training dataset is helpful. To train a stable and reliable network, the training dataset is expected to cover a large population with a variety of age, sex, weight, races, types of diseases, and covers typical clinical scenarios such as acquisition time and injected activity. Moreover, the learning target is expected to have the lowest image noise and the highest image contrast. To achieve this goal, the acquisition time should be long enough to collect adequate counts. It is very hard for the conventional PET/CT scanners because of their limited sensitivity. Benefits from its ultra-high sensitivity, total-body or long axial field-of-view (LAFOV) PET/CT scanners such as uEXPLORER [37], PennPET [38] and Biograph Vision Quadra [39] have demonstrated extraordinary image quality than conventional PET/CT scanners. So if the training data are from the total-body or LAFOV PET/CT scanners, the network performance may be greatly improved. Thirdly, the denoising step could be integrated into the iterative reconstruction algorithm, not just work after the image reconstruction. Contrast loss is inevitable when post-processing the high noise images. This is because the small lesions are almost overwhelmed by the background noise. The signal features extracted by the network are too weak to be differed from the noise. To tackle this problem, combination of iterative reconstruction algorithm and neural networks has been actively studied [39–42]. This may inspire the development of deep learning methodologies with both reduced image noise and improved image contrast.

This study has several limitations. It was a single-centre study with limited retrospective data from one PET/CT system. Further studies on a larger number of patients in a clinical setting using different PET/CT systems may be required. The cohort of overweight and obese patients enrolled in this study was small, which may introduce bias into quantitative analyses for this group of our study population. Detectability of small lesions is affected by many factors, e.g. lesion size, uptake, shape, location, respiratory motion, and, most importantly, the acquisition time. The analysis of statistical fluctuations of reduced acquisition time in small lesion measurements is necessary, but it is beyond the scope of this study. This study used shorted acquisition time to simulate the scenario of reduced activity, and future study can be performed with a real-world reduced injected activity to validate our findings. Up to now, HYPER DLR method has only been trained with 18F-FDG PET images, and thus can only be applied to 18F-FDG PET applications. However, PET imaging with other tracers, such as prostate specific membrane antigen (PSMA) and Fibroblast activation protein inhibitor (FAPI), is becoming more widely used. The HYPER DLR can be applied in other tracers if the network is trained in the future.

## Conclusion

In PET/CT oncological images used in this study, the developed deep learning algorithm HYPER DLR showed improvements in image quality compared to that post-processed by Gaussian filter. HYPER DLR reconstruction yielded improved results in liver SNR even if the acquisition duration was shortened to 33.3% of that in the reference group, indicating the superior noise reduction of this approach. In addition, HYPER DLR can generate images with uncompromised lesion contrast with an acquisition duration shortened by 25–50%. Thus, it offers potential applications in PET imaging with reductions in required FDG activity or acquisition time.

## Supplementary Information

**Additional file 1. Fig. S1**: Liver metastasis of a 74-year-old female with gallbladder carcinoma. The lesion (red arrow) was identified in the reference OSEM_180 images with a measured SUVmax of 3.64 (left) but missed in the DLR_90 and DLR_60 images due to the high image noise and respiratory motion artefacts. SUV, standardized uptake value.

**Additional file 2. Fig. S2**: The lesion with the maximum TsBR value among all missed lesions was found to be a pelvic metastatic lymph node of a 66-year-old male with bladder cancer. It was challenging to distinguish the lesion with a diameter of 6.1 mm from the physiological uptake observed in the intestinal tract (line intersection, right). TsBR, tumour-to-surrounding-background ratio.

## Data Availability

Data are available on request to the corresponding author.
