# Supplementary material for: Deep learning-assisted PET imaging achieves fast scan/low-dose examination
Source: EJNMMI Phys. 2022 Feb 4;9:7. doi: 10.1186/s40658-022-00431-9 (PMC8816983; doi:10.1186/s40658-022-00431-9)
Supplement: Supplementary file 1 — Additional file 1. Fig. S1: Liver metastasis of a 74-year-old female with gallbladder carcinoma. The lesion (red arrow) was identified in the reference OSEM_180 images with a measured SUVmax of 3.64 (left) but missed in the DLR_90 and DLR_60 images due to the high image noise and respiratory motion artefacts. SUV, standardized uptake value. [file 40658_2022_431_MOESM1_ESM.docx]

## Supplementary material 1

**Deep learning assisted PET imaging achieves fast scan/low-dose examination**

Yan Xing^*1^, Wenli Qiao^*1^, Taisong Wang^1^, Ying Wang^2^, Chenwei Li^2^, Yang Lv^2^, Chen Xi^2^, Shu Liao^3^, Zheng Qian^2^, Jinhua Zhao^1^

*^1^Department of Nuclear Medicine, Shanghai General Hospital, Shanghai Jiaotong University School of Medicine, Shanghai, People's Republic of China,*

*^2^United Imaging Healthcare, Shanghai, People's Republic of China,*

*^3^Shanghai United Imaging Intelligence Co. Ltd, Shanghai, People's Republic of China*

Corresponding author: Jinhua Zhao, Department of Nuclear Medicine, Shanghai General Hospital, Shanghai Jiaotong University School of Medicine, No. 100 Haining Road, Shanghai, 200080, People's Republic of China*.*

E-mail: [zhaojinhua1963@126.com](mailto:zhaojinhua1963@126.com)

*Contributed equally to this work


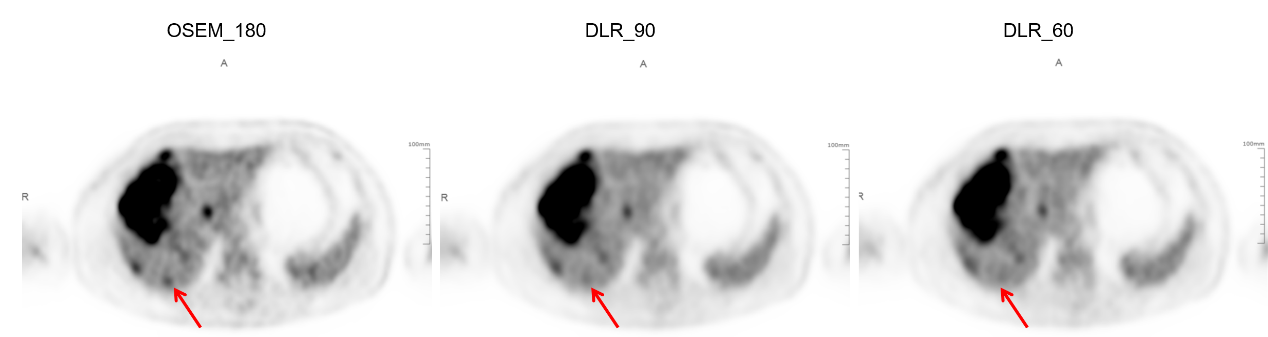


**Supplementary Fig. S1** Liver metastasis of a 74-year-old female with gallbladder carcinoma. The lesion (red arrow) was identified in the reference OSEM_180 images with a measured SUVmax of 3.64 (left) but missed in the DLR_90 and DLR_60 images due to the high image noise and respiratory motion artefacts. SUV, standardized uptake value
