# Supplementary material for: Deep learning-assisted PET imaging achieves fast scan/low-dose examination
Source: EJNMMI Phys. 2022 Feb 4;9:7. doi: 10.1186/s40658-022-00431-9 (PMC8816983; doi:10.1186/s40658-022-00431-9)
Supplement: Supplementary file 2 — Additional file 2. Fig. S2: The lesion with the maximum TsBR value among all missed lesions was found to be a pelvic metastatic lymph node of a 66-year-old male with bladder cancer. It was challenging to distinguish the lesion with a diameter of 6.1 mm from the physiological uptake observed in the intestinal tract (line intersection, right). TsBR, tumour-to-surrounding-background ratio. [file 40658_2022_431_MOESM2_ESM.docx]

## Supplementary material

**Deep learning assisted PET imaging achieves fast scan/low-dose examination**

Yan Xing^*1^, Wenli Qiao^*1^, Taisong Wang^1^, Ying Wang^2^, Chenwei Li^2^, Yang Lv^2^, Chen Xi^2^, Shu Liao^3^, Zheng Qian^2^, Jinhua Zhao^1^

*^1^Department of Nuclear Medicine, Shanghai General Hospital, Shanghai Jiaotong University School of Medicine, Shanghai, People's Republic of China,*

*^2^United Imaging Healthcare, Shanghai, People's Republic of China,*

*^3^Shanghai United Imaging Intelligence Co. Ltd, Shanghai, People's Republic of China*

Corresponding author: Jinhua Zhao, Department of Nuclear Medicine, Shanghai General Hospital, Shanghai Jiaotong University School of Medicine, No. 100 Haining Road, Shanghai, 200080, People's Republic of China*.*

E-mail: [zhaojinhua1963@126.com](mailto:zhaojinhua1963@126.com)

*Contributed equally to this work


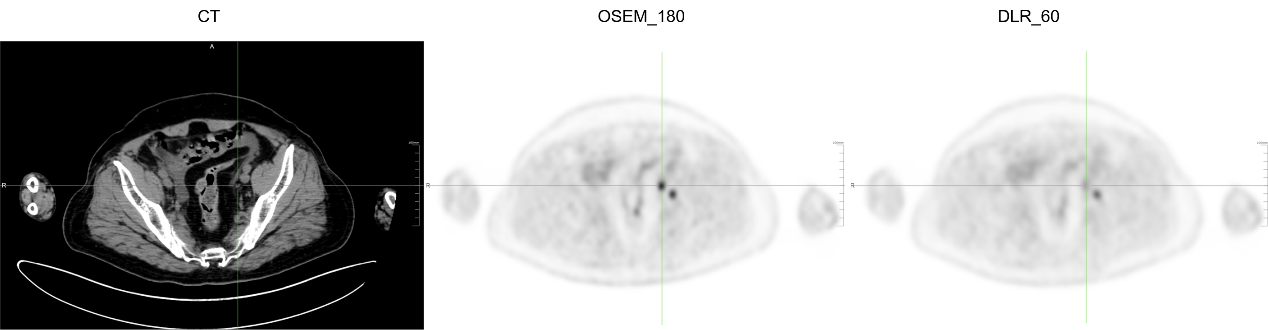


**Supplementary Fig. S2** The lesion with the maximum TsBR value among all missed lesions was found to be a pelvic metastatic lymph node of a 66-year-old male with bladder cancer. It was challenging to distinguish the lesion with a diameter of 6.1 mm from the physiological uptake observed in the intestinal tract (line intersection, right). TsBR, tumour-to-surrounding-background ratio
